# The impact of exercise on mental health during the COVID-19 pandemic: a systematic review and meta-analysis

**DOI:** 10.3389/fpubh.2023.1279599

**Published:** 2023-10-04

**Authors:** Chaochao Wang, Zuguo Tian, Qiaoyou Luo

**Affiliations:** Department of Physical Education, Hunan University, Changsha, Hunan, China

**Keywords:** COVID-19, exercise, physical activity, mental health, psychological health

## Abstract

**Introduction:**

Amidst the COVID-19 pandemic, there has been a global surge in mental health challenges. This study (PROSPERO CRD42023443860) aimed to investigate the impact of exercise on individuals’ mental health through systematic evaluation and meta-analysis to develop a scientific exercise program.

**Methods:**

We systematically searched the literature up to August 2023 using PubMed, Web of Science, and SCOPUS. The Cochrane Risk Assessment Tool gaged the methodological quality of the included literature.

**Results:**

Among the initially identified 10,343 search records, 12 studies were deemed to meet the criteria for systematic review and meta-analysis. Exercise significantly improved anxiety (SMD -0.81, 95% CI -1.10 to −0.52, *p*  < 0.00001), depression (−1.02, 95% CI -1.42 to −0.62, *p*  = 0.0001), stress (−1.05, 95% CI -1.33 to −0.78, *p*  < 0.00001), and quality of life (1.11, 95% CI 0.81 to 1.41, *p*  < 0.00001). Subgroup analyzes indicated that a single exercise session lasting 30–40 min had the most pronounced effect on reducing anxiety (−1.29, 95% CI -1.12 to −0.86, *p*  < 0.00001) and depression (−1.76, 95% CI -2.24 to −1.28, *p*  < 0.00001). Similarly, an exercise frequency of 3–5 times per week yielded the greatest benefits for anxiety (−1.31, 95% CI -2.15 to −0.46, *p*  < 0.00001) and depression (−1.27, 95% CI -2.11 to −0.41, *p*  = 0.0003). Notably, exercise exhibited its most significant impact on depression improvement in the 40–64 age group (−1.32, 95% CI -1.78 to −0.86, *p*  < 0.00001). Moreover, exercise notably enhanced anxiety levels among individuals in middle and upper-income brackets (−0.99, 95% CI -1.11 to −0.87, *p*  < 0.00001).

**Conclusion:**

Exercise alleviated anxiety disorders, depression, stress levels, and quality of life during the COVID-19 pandemic. The most significant impact on anxiety and depression improvement is achieved by engaging in 30–40 min of exercise sessions, 3–5 times per week.

## 1. Introduction

The COVID-19 pandemic, caused by the novel coronavirus, has rapidly spread on a global scale, prompting widespread public health crises (1). In the face of such public health crises, numerous countries have advocated for minimizing direct social interactions and implementing lockdown measures to curb the virus’s transmission. However, these stringent measures, combined with the inherent fear surrounding the virus, can induce significant psychological strain and distress. These may encompass concerns about physical well-being, heightened apprehension regarding economic stability, and even the emergence of anxiety and depression due to the enforced restrictions (2). Statistical analysis reveals that the prevalence of depression and anxiety disorders on a global scale throughout the COVID-19 pandemic stands at 24.0 and 21.3%, respectively (3). These psychological challenges transcend age demographics and profoundly affect individuals’ mental well-being.

Psychological issues are typically treated through either medication or psychological intervention. Medication serves as a prevalent approach for managing anxiety disorders, involving the utilization of GABA receptor agonists and benzodiazepines (4, 5), as well as depression, employing medications such as selective serotonin reuptake inhibitors (SSRIs) and norepinephrine reuptake inhibitors (SNRIs) (6, 7). However, it is essential to note that the effectiveness of these medications varies due to the diverse nature of psychological conditions. Moreover, it is worth acknowledging that medications can potentially compromise neuronal function, resulting in an array of somatic symptoms and adverse impacts on bone metabolism (8). Furthermore, a range of psychotherapies, encompassing positive thinking therapy, hypnotherapy, music therapy, and Morita therapy, are integrated into the treatment paradigm. These psychotherapeutic approaches complement traditional treatments and play a crucial role in ameliorating psychological problems. Nonetheless, it is important to consider that the duration of psychotherapeutic interventions is often limited that outcomes exhibit variability among individuals (9). In conclusion, these governance strategies have not yet achieved optimal outcomes.

An increasing number of trials and studies are concentrating on exercise. Several studies have demonstrated a substantial influence of exercise on mental health (10–13). Furthermore, it has been established as both an effective and cost-efficient intervention for a wide spectrum of psychological issues, encompassing anxiety disorders and depression (14, 15). Scholarly research has deduced a notable correlation between physical activity and reduced occurrences of mental ailments (such as depression, stress, negative emotions, and overall psychological distress) and heightened psychological well-being (including self-image, life satisfaction, well-being, and mental health) (16–18). Moreover, the COVID-19 pandemic has triggered extensive psychological challenges. Over this specific period, numerous studies have indicated that exercise can ameliorate COVID-19-related negative psychology. For instance, one study demonstrated that practicing tai chi during the pandemic effectively intervened to enhance psychological and physical functioning among older adults (19). Similarly, interventions involving yoga and mindfulness interventions have exhibited a significant impact on alleviating anxiety and depression symptoms in adolescents in middle schools (20). Nevertheless, these studies were limited in sample size, and the effects of exercise on mental health displayed variability. Further incorporation of additional studies is imperative to achieve comprehensive representation. In the first place, we posit that physical exercise exerts a positive influence on psychological well-being, implying a significant positive correlation between engaging in physical activities and mental health. Secondly, we hypothesize that exercise can diminish the severity of symptoms related to anxiety, depression, and stress while also enhancing the overall quality of life, thus mitigating the adverse impacts of mental disorders. Thirdly, considering the unique psychological stresses during the COVID-19 pandemic, we postulate that exercise may have a more pronounced impact on mental health during this period, as it can serve as an effective psychological intervention. Fourthly, we also propose that different forms of exercise, such as Tai Chi, yoga, and mindfulness interventions, may yield varying effects on mental health, necessitating further comparisons of their outcomes. Therefore, a comprehensive systematic review and meta-analysis are required to obtain more precise conclusions. These hypotheses are expected to guide the design and analysis of research, enabling a deeper understanding of the impact of exercise on mental health during the COVID-19 pandemic.

## 2. Methods

This systematic review was registered with PROSPERO, the International Prospective Registry of Systematic Reviews (registration number: CRD42023443860). The review was conducted following the guidelines outlined in the Preferred Reporting Items for Systematic Reviews and Meta-Analysis (PRISMA) statement (21).

### 2.1. Literature search strategy

Two independent researchers systematically conducted an extensive search of randomized controlled trials (RCTs) across the PubMed, Web of Science, and Scopus databases. RCTs published in English before August 1, 2023, that investigated the impact of exercise on mental health during the COVID-19 pandemic were eligible for inclusion. Additionally, reference tracking of published trials and meta-analytic reviews in the field was performed to ensure the comprehensive inclusion of all relevant studies. The search employed specific terms as follows: “CoV-19” OR “SARS-CoV-2” OR “2019 nCoV,” “Physical Activity (PA),” “Exercise” OR “Physical Activity,” and “Mental Health” OR “Mental Hygiene.” Detailed information on the search strategy can be found in Supplementary material.

### 2.2. Eligibility criteria

The inclusion criteria were established following the PICOS method (population, intervention, comparison, outcome, and study design). Studies were eligible for inclusion if they met the following criteria: (1) Population: all groups during the COVID-19 epidemic, encompassing specific cohorts of COVID-19-infected individuals, convalescents, and healthcare workers. And the search was not limited to any particular population to ensure comprehensive meta-analyzes. (2) Interventions: we considered various types of exercises, such as aerobic workouts, strength training, yoga, and more. No specific requirements were set for intervention frequency, intensity, or duration. Interventions were categorized as single- or multiple-group interventions. (3) Control: the control group either did not receive interventions, received non-exercise interventions, or received routine care without medical treatment. (4) Outcome: based on the Isabel Dore classification (22), mental health components encompass emotional health, quality of life, and psychological and social well-being. This systematic review focused on depression, anxiety, perceived stress, and quality of life. (5) Study Design: Only English-language, peer-reviewed RCTs were included. Reviews, single-case studies, dissertations, conference papers, and abstracts were excluded.

Exclusion criteria were as follows (1): Reviews, letters, editorial comments, case reports, conference abstracts, unpublished articles, and non-English articles were excluded. (2) Studies without quantified results or lacking corresponding outcome indicators were excluded. (3) Literature inaccessible in full text through various channels and methods was excluded. (4) Articles of poor research quality or lacking obtainable qualitative information were excluded. (5) Literature lacking a control group was excluded.

### 2.3. Study selection and data extraction

All retrieved literature was imported into the EndNote software for de-duplication. Subsequently, two researchers (CCW and QYL) independently screened the titles, abstracts, and full texts of the literature. In cases of disagreement, a consensus was reached with the assistance of a third researcher to determine the final results. Following the literature screening, the two researchers utilized a Microsoft Excel spreadsheet for information extraction and coding from the trials. Information extracted for each trial encompassed the first author, country, publication year, study population, intervention details, intervention protocol (including duration of single intervention, intensity, and duration of intervention), measurement tools, and outcome indicators.

### 2.4. Quality appraisal

The Cochrane Risk of Bias tool was employed to evaluate the quality of eligible trials, with a focus on seven aspects: (i) randomized sequence generation; (ii) allocation concealment; (iii) blinding of participants and personnel; (iv) blinding of outcome assessment; (v) completeness of outcome data; (vi) selective reporting of study results; and (vii) other sources of bias. Each study was assessed holistically based on a 6-item criteria set, then categorized into three levels: low risk of bias, moderate risk of bias, and high risk of bias. Risk of bias diagrams were generated using Review Manager 5.3 software. The quality assessment was conducted independently by two researchers (CCW and QYL), and any discrepancies were resolved through discussions with a third individual (ZGT).

### 2.5. Data synthesis and analysis

The evidence synthesis was conducted using Review Manager version 5.3, provided by the Cochrane Collaboration Network, located in Oxford, United Kingdom. The variables under investigation included anxiety, depression, perceived stress, and quality of life, all of which were analyzed as continuous variables. The results for all indicators are presented as 95% confidence intervals (CIs). Heterogeneity within the studies was evaluated utilizing both the chi-square (Χ^2^) test (Cochran’s Q) and the inconsistency index (*I*^2^) (23). Significant heterogeneity was determined by Χ^2^ *p*-values <0.05 or I^2^ > 50%. In cases of significant heterogeneity, a random effects model was employed; otherwise, a fixed effects model was used. Funnel plots were generated using Review Manager version 5.3 from the Cochrane Collaboration in Oxford, United Kingdom. These plots were analyzed in a minimum of two of the included randomized controlled trials (RCTs). Subgroup analysis was undertaken for analyzes exhibiting high heterogeneity, focusing on diverse study characteristics to identify potential sources of heterogeneity. To ensure the reliability of the findings, sensitivity analyzes were performed, wherein each article was individually removed to assess its impact on the combined effect. If the trial group encompassed 10 or more trials, the potential for publication bias was assessed through the Begg and Egger tests using STATA 12.0, developed by Stata Corp in College Station, TX, United States. A significance level of *p* < 0.05 was considered indicative of statistically significant publication bias.

## 3. Results

### 3.1. Study selection

**Figure 1:**
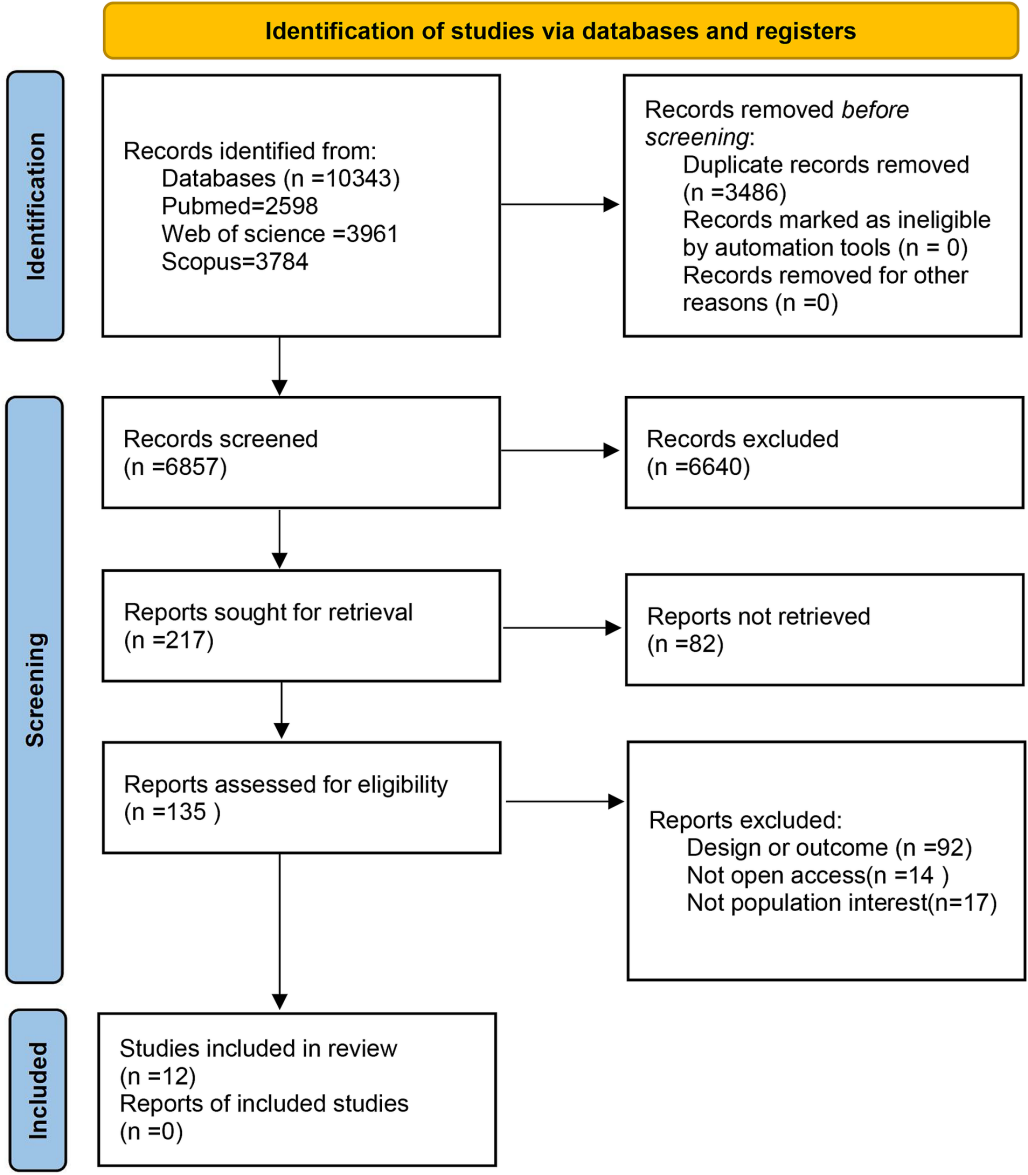
PRISMA 2020 flow diagram.

Initially, 10,343 studies were identified through searches conducted in the three databases. Following the elimination of 3,486 duplicates, the screening of titles and abstracts yielded 135 full-text manuscripts for assessment. After a thorough evaluation of these full texts, 123 articles were subsequently excluded. Ultimately, 12 articles fulfilled the stipulated criteria and were incorporated into our systematic review and meta-analysis of the collected studies (see Figure 1).

### 3.2. Study characteristics

Table 1 displays the primary characteristics of the participants and interventions. The included studies span the years 2020 to 2023, comprising a total of 12 randomized controlled trials (RCTs) (Table 1). Within these 12 studies, the participant pool consisted of 1,689 individuals, with 919 (54.4%) being male. The study encompassed participants from nine different countries. Among these studies, three studies (25%) were conducted in China (24–26), one study (8.3%) in Lithuania (19), one study (8.3%) in the United Kingdom (27), two studies (16.7%) in Turkey (28, 33), one study (8.3%) in Iran (29), one study (8.3%) in Tunisia (29), one study (8.3%) in the United States (20), one study (8.3%) in South Korea (31), and one study (8.3%) in Saudi Arabia (32).

**Table 1:** Characteristics of studies included in this meta-analysis.

Study	Country	Sample size	Sex (Male, n, %)	Age (M ± SD)	Intervention	Intensity	Duration of a single intervention	Outcome measures	Duration of Intervention (week)	Outcome
Liu et al. (24) RCT	China	IG: 36 CG: 36	IG: 24(66.7%) CG: 25(69.4%)	IG:69.4 (8.0) CG: 68.9 (7.6)	IG: Home exercise CG: No exercise intervention	N/A	10 min per day	Self-rating depression scale self-rating anxiety scale the Short Form-36	6	Depression* Anxiety* Quality of life* (IG)
Liu et al. (25) RCT	China	IG: 25 CG:26	IG: 14(56.00%) CG: 14(53.85%)	50.41 ± 13.04	IG: Progressive muscle relaxation exercises CG: No exercise intervention	N/A	30 min per day	The Spielberger State–Trait Anxiety Scale	1	Anxiety* (IG)
Solianik et al. (19) RCT	Lithuania	IG:15 CG:15	4(13.33%)	67.0 ± 5.9	IG: Tai chi CG: No exercise intervention	N/A	a biweekly 60-min	Perceived Stress Scale Hospital Anxiety and Depression Scale	10	Depression*(I) Perceived Stress* (IG&CG)
Zheng et al. (26) RCT	China	IG:485CG:469	IG:248 (51.1%) CG:251 (53.5%)	IG:13.5 ± 0.50 CG:13.5 ± 0.50	IG: health education + broadcast exercise programs CG: only health education	N/A	15 min each time; 4 times per day	Spence Children’s Anxiety Scale	2	Anxiety* (IG)
Wadhen et al. (27) RCT	UK	IG:17 CG:17	IG:0(0%) CG:3(17.65%)	IG:42.7 ± 10.94 CG:42.2 ± 10.20	IG: yoga CG: No exercise intervention	N/A	a minimum of two and a maximum of three 50-min classes each week for six weeks	Perceived stress Depression, Anxiety & Stress scale; DASS-21 WEMWBS	6	Depression* Perceived Stress* Anxiety* Quality of life* (IG)
Ozlu et al. (28) RCT	Turkey	IG:33 CG:34	IG:21(63.64%) CG:16(47.06%)	IG:36.48 ± 11.63 CG:33.15 ± 11.90	IG: progressive muscle relaxation exercises CG: No exercise intervention	N/A	twice a day for 5 days 20–30 min	The State–Trait Anxiety Inventory	1	Anxiety* (IG)
Zendehdel et al. (29) RCT	Iran	IG:57 CG:58	IG:57(100%) CG:58(100%)	IG:22.93 ± 1.72 CG:28.12 ± 1.71	IG: Progressive muscle relaxation exercises CG: No exercise intervention	N/A	Three times a week; 30 min each time	Corona disease anxiety scale	4	Anxiety* (IG)
Baklouti et al. (30) RCT	Tunisia	IG:65 CG:95	IG:42(42.86%) CG:56(57.14%)	65–85 (Range)	IG: yoga CG: No exercise intervention	N/A	80 twice a week	Depression, Anxiety, and Stress Scales	8	Depression* Perceived Stress* Anxiety* (IG)
Bazzano et al. (20) RCT	USA	IG:42 CG:44	IG:23(57.5%) CG:21(50.0%)	11–14 (Range)	IG: yoga CG: No exercise intervention	N/A	45 once a week	the Screen for Child Anxiety-Related Disorders the Patient Health Questionnaire revised for adolescents	8	Depression* (IG)
Kim et al. (31) RCT	Korea	IG:8 CG:8	IG:8(100%) CG:8(100%)	IG:38.14 ± 1.39 CG:39.71 ± 2.01	IG: Online Pilates Exercise CG: No exercise intervention	50–60% of the maximum heart rate,	twice a week, 50 min a day.	Edinburgh Postnatal Depression Scale Perceived Stress Scale	8	Depression* Perceived Stress* (IG)
Ibrahim et al. (32) RCT	Saudi Arabia	MIG:24 LIG:24 CG:24	MIG:11(45.83%) LIG:12(50%) CG:8(33.33%)	MIG:62.6 ± 5.01 LIG:62.5 ± 4.67 CG:62.7 ± 4.3	IG: Aerobic training CG: No exercise intervention	moderate-intensity low-intensity	40 min four times a week	Hamilton Anxiety and Depression Scale SF-36	10	Depression* Anxiety* Quality of life* (IG)
Gunebakan et al. (33) RCT	Turkey	IG:16 CG:16	IG:16(100%) CG:16(100%)	IG:25.56 ± 4.55 CG:29.81 ± 7.00	IG: yoga CG: No exercise intervention	N/A	Twice a week for 45 min	Beck Depression Scale The State and Trait Anxiety Inventory	6	Depression* Anxiety* (IG)

EG experimental group. IG control group. MIG Moderate intensity group. LIG Low-intensity group. *significant improvement.

Regarding exercise interventions, the chosen approaches varied. One study (8.3%) implemented home exercise (24), three studies (25%) incorporated progressive muscle relaxation exercises (25, 28, 29), another study (8.3%) adopted Tai chi (19), and a separate one (8.3%) utilized broadcast exercise programs (26). Additionally, four studies (33.3%) integrated yoga (20, 27, 30, 33), Additionally, four studies (33.3%) integrated yoga (31), and one study (8.3%) implemented aerobic training (32). Within our review, only a single study reported the intensity of the intervention, with intervention frequency showing substantial variation. Exercise intervention durations ranged from 1 to 10 weeks, with the majority lasting 6 weeks (25%) or 8 weeks (25%).

Regarding the intervention outcomes, anxiety was addressed in 11 studies (91.7%) (19, 20, 24–30, 32, 33), depression in 8 studies (66.7%) (19, 20, 24, 27, 30–33), perceived stress in 4 studies (33.3%) (19, 27, 30, 31), and quality of life in three studies (25%) (24, 27, 32). Although various validated scales were employed across trials for outcome assessment, the data collection process was consistently overseen by experienced staff. For assessing anxiety, instruments included the Self-Rated Anxiety Scale (24, 26), Spielberger State–Trait Anxiety Scale (25, 28, 33), DASS-21 (27, 30), Corona disease anxiety scale (29), HADS (19, 32), and SCARED (20). The assessment of depression employed instruments such as SDS (24), PSS-10 (19, 26), DASS-12 (30), PHQA (20), EPDS (31), HADS (32), and BDS (33). The evaluation of perceived stress utilized the PSS-10 (19, 27, 31), and DASS-21 (30), while the quality of life was assessed using the SF-36 (24), WEMWBS (27), and SF-36 (32).

### 3.3. Risk-of-bias assessment

**Figure 2:**
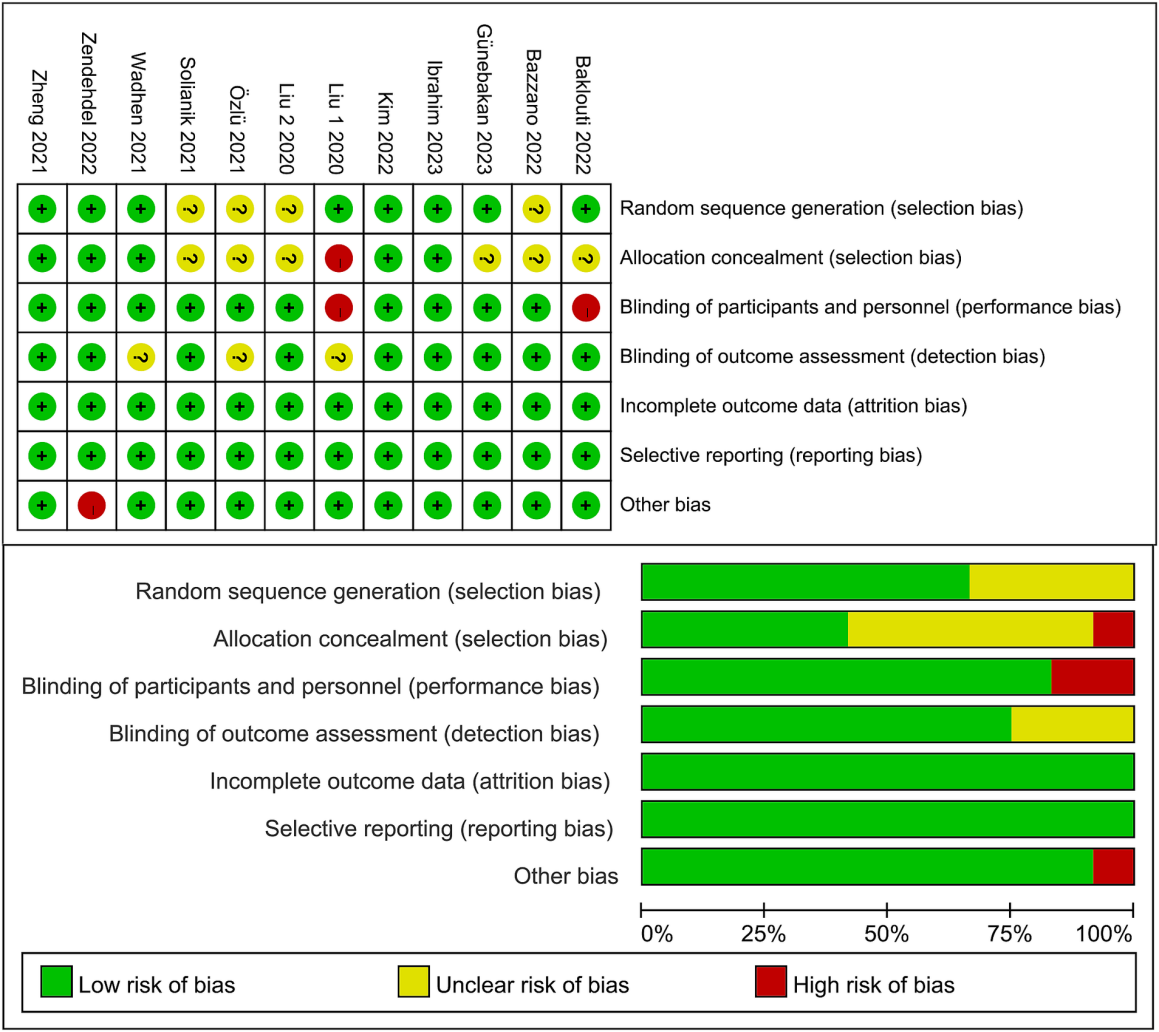
Risk-of-bias summary. Above: Risk of bias summary: review authors’ judgments about each risk of bias item for each included study. Below: Risk of bias graph: review authors’ judgments about each risk of bias item presented as percentages across all included studies.

(Figure 2) summarizes the risk of bias. Overall, the risk of bias in the 12 trials included in the review was within acceptable limits. Random sequence was adequately determined in 8 trials (66.7%), and allocation concealment was adequately recognized in 5 trials (41.7%). Ten trials (83.3%) blinded participants and staff. Nine trials (75%) were blinded to the outcome assessor, and the risk of detection bias for these trials was judged to be low. In 12 trials, no dropouts or selectivity were reported. Among the other risks of bias, only one mentioned interference by other factors. Therefore, the risk of reporting bias for these trials was rated as low.

### 3.4. Results of the meta-analysis

In the trials included, various instruments were utilized to assess mental health outcomes during the COVID-19 pandemic. Our review primarily focused on conducting meta-analyzes for anxiety, depression, perceived stress, and quality of life. The efficacy analysis was based on the change from baseline to final value scores. The outcomes of our analysis for each of these aspects are presented below.

#### 3.4.1. Anxiety

**Figure 3:**
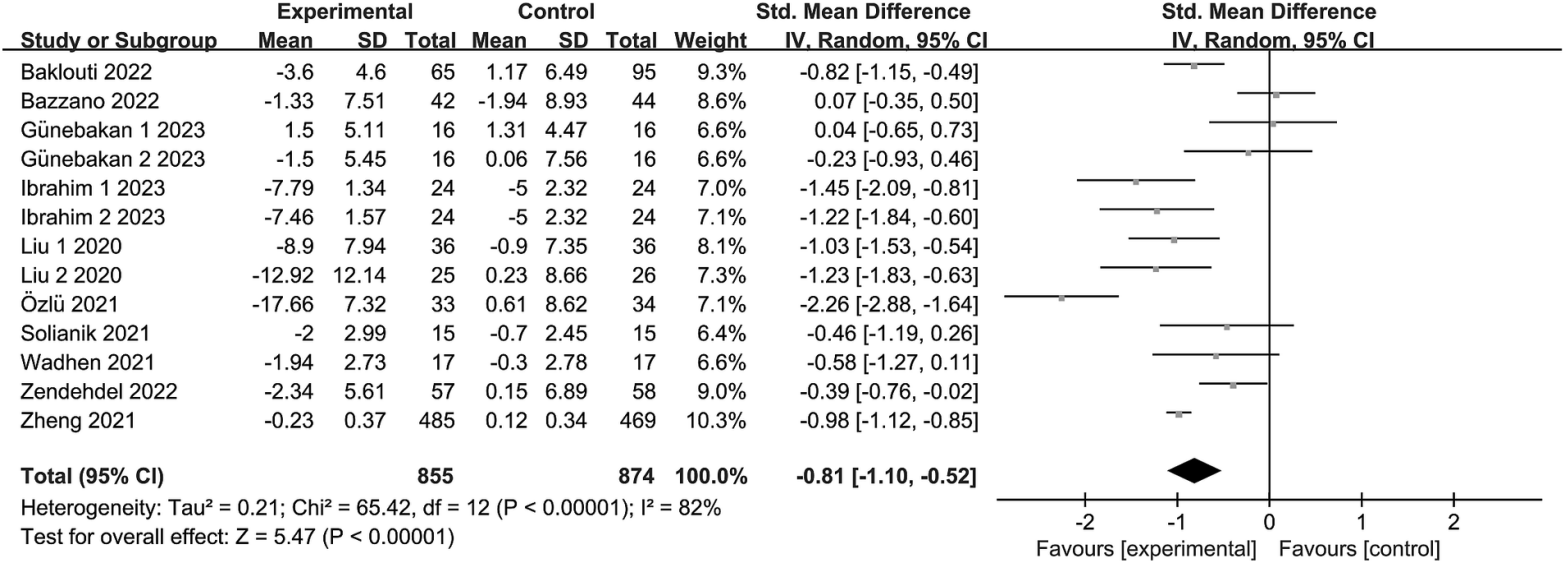
Forest plot for Exercise on Anxiety. CI, confidence interval.

Eleven trials (19, 20, 24–30, 32, 33) reported anxiety, involving a total of 1,673 subjects. In one study (32), the intervention group was subdivided into two segments, each with varying intensities. Another study (33) presented two distinct anxiety outcomes (STAI-I and STAI-II). Consequently, the meta-analysis encompassed 13 items. Given the observed heterogeneity in this review (*I*^2^ = 82%, *p* < 0.00001), a random effects model was adopted. The findings revealed an aggregated sample size of 1729, accompanied by substantial evidence indicating significant levels of anxiety due to exercise intervention in comparison to the control group [SMD = −0.81, 95% CI = (−1.10, −0.52), *p* < 0.00001; see Figure 3].

#### 3.4.2. Depression

**Figure 4:**
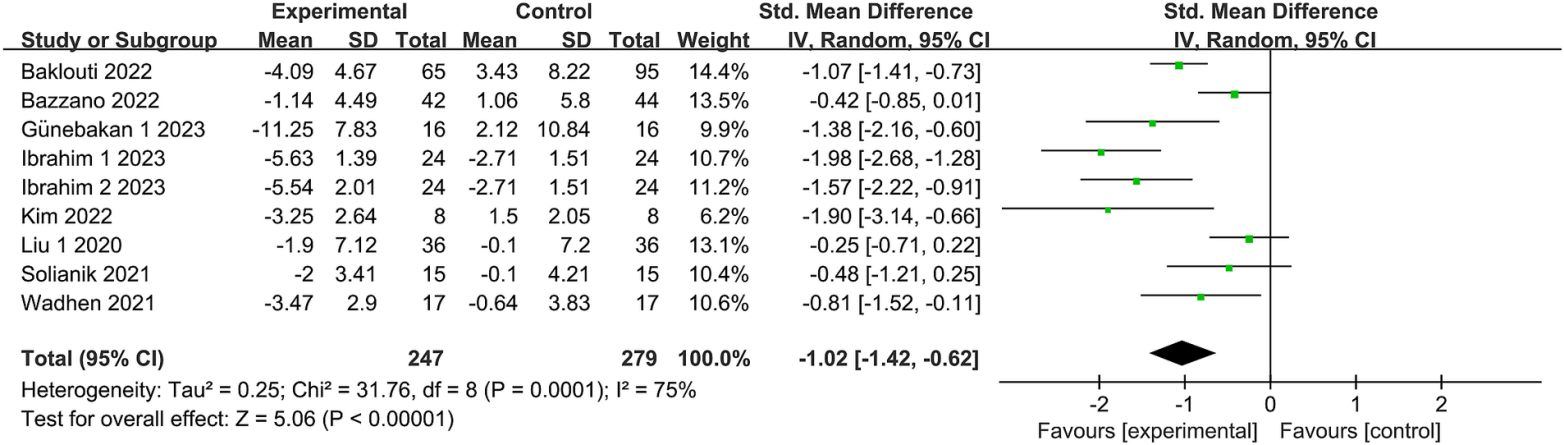
Forest plot for Exercise on Depression. CI, confidence interval.

Eight studies (19, 20, 24, 27, 30–33) reported depression, involving a total of 502 subjects. One study (32) subdivided the intervention group into two segments with differing intensities. Nine studies were included in the meta-analysis. Owing to the heterogeneity in this review (*I*^2^ = 75%, *p* = 0.0001), a random effects model was employed. There was substantial evidence indicating a significant distinction in depression levels between the exercise intervention and control groups [SMD = −1.02, 95% CI = (−1.42, −0.62), *p* < 0.00001; see Figure 4].

#### 3.4.3. Stress

**Figure 5:**
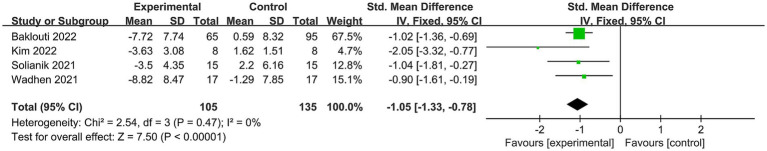
Forest plot for Exercise on Perceived Stress. CI, confidence interval.

Stress was documented in four trials (19, 27, 30, 31), involving 240 subjects. As no heterogeneity was observed in these findings (*I*^2^ = 0%), we employed a fixed effects model to combine the results. The overall outcome indicated a notable disparity in perceived stress between the exercise intervention and control groups, with significant statistical significance [SMD = −1.05, 95% CI = (−1.33, −0.78), *p* < 0.00001; see Figure 5].

#### 3.4.4. Quality of life

**Figure 6:**
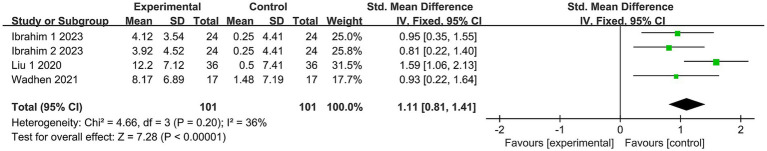
Forest plot for Exercise on Quality of Life. CI, confidence interval.

The impact of exercise on quality of life was assessed in three trials (24, 27, 32), encompassing 202 subjects. Due to the absence of heterogeneity in this analysis (*I*^2^ = 36%), a fixed-effects model was selected. The study outcomes demonstrated a substantial effect of exercise on the quality of life in comparison to the control group [SMD = 1.11, 95% CI = (0.81, 1.41), *p* < 0.00001; see Figure 6].

### 3.5. Sensitivity analysis

**Figure 7:**
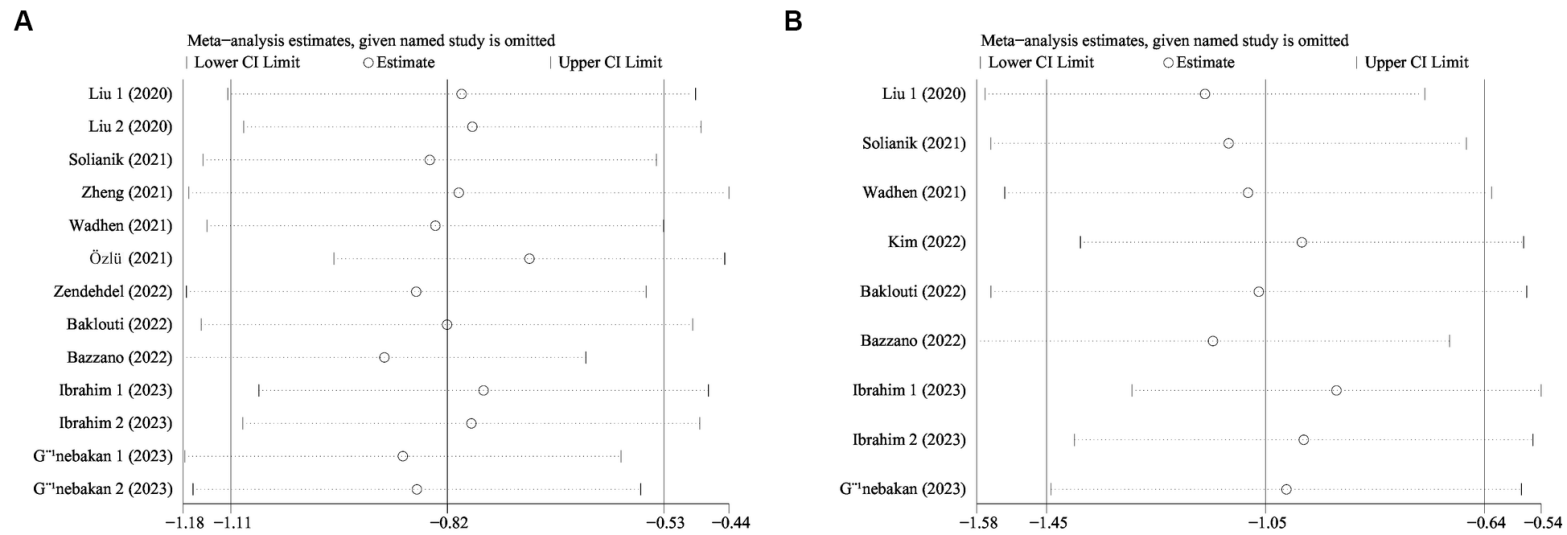
Sensitivity analysis of **(A)** Anxiety **(B)** Depression.

We conducted sensitivity analyzes to evaluate the impact of each study on exercise intervention, anxiety, and depression by systematically removing studies on a case-by-case basis (see Figure 7A). The results of the meta-analysis concerning exercise intervention for anxiety yielded a statistically significant outcome with an odds ratio (OR) of −0.82 (95% CI: −1.11, −0.53; see Figure 7B). Similarly, the meta-analysis on exercise interventions for depression also demonstrated statistically significant results, with an OR of −1.05 (95% CI: −1.45, −0.64). Our sensitivity analyzes exhibited the robustness of the findings regarding exercise interventions for anxiety disorders and depression, even after excluding individual studies.

### 3.6. Publication risk of bias detection

**Figure 8:**
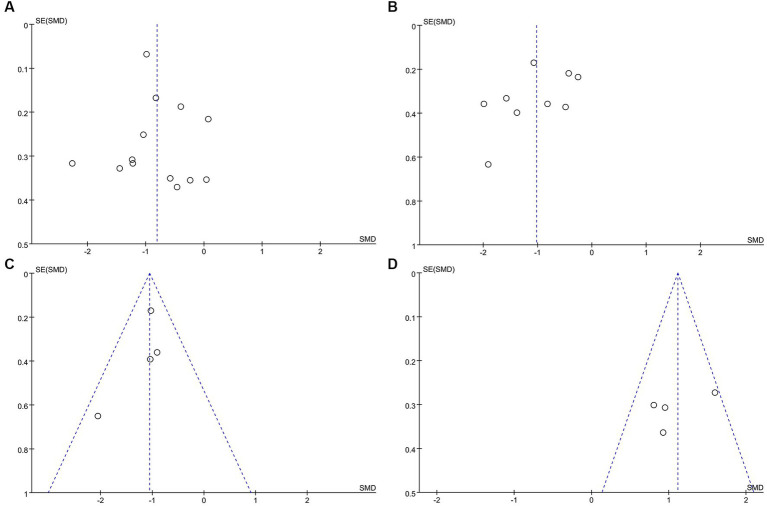
Funnel plot. **(A)** Funnel plot for Anxiety. **(B)**Funnel plot for Depression. **(C)** Funnel plot for Stresses. **(D)** Funnel plot for Quality of life.

The funnel plot depicting the impact of exercise on anxiety, depression, stress, and quality of life (see Figure 8) illustrates a symmetrical distribution on both its left and right sides, indicating minimal publication bias in the results.

**Figure 9:**
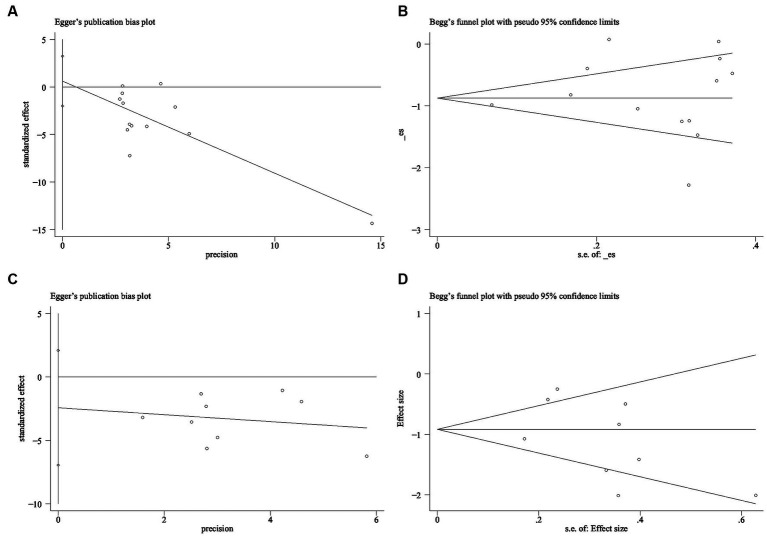
Publication bias detection **(A)** Anxiety Egger test **(B)** Anxiety Begg test **(C)** Depression Egger test **(D)** Depression Begg test.

More than ten studies have examined indicators of both anxiety and depression. To assess publication bias, we employed the Egger and Begg linear regression tests (refer to Table 2). Anxiety (*t* = 0.52, *p* = 0.615; Figure 9A) and depression (*t* = −1.27, *p* = 0.244; Figure 9B) were evaluated using Egger’s linear regression, demonstrating no significant difference between the two conditions (*p* > 0.05). The Egger linear regression analysis indicated the absence of publication bias. Begg’s linear regression yielded anxiety (z = 0.55, Pr = 0.583; Figure 9C) and depression (*z* = 0.73, Pr = 0.466; Figure 9D) values. Similarly, no significant difference (*p* > 0.05) between anxiety and depression was observed. The Begg linear regression also confirmed the absence of publication bias in these measures.

**Table 2:** analysis of bias.

Outcomes	Egger		Begg	
	*t*	*P*	*z*	Pr
Anxiety	0.52	0.615	0.55	0.583
Depression	−1.27	0.244	0.73	0.466

### 3.7. Subgroup analysis

The results indicated no significant heterogeneity for stress (*I*^2^ = 0%) and low heterogeneity for quality of life (*I*^2^ = 36%), implying consistent findings across the included studies and a limited study pool. As a result, subgroup analyzes were not conducted for stress and quality of life outcomes. Conversely, our findings demonstrated significant heterogeneity for anxiety disorders (*I*^2^ = 82%) and depression (*I*^2^ = 75%), prompting subgroup analyzes for both conditions. Subgroup analyzes were employed for the five components of the exercise intervention program in the meta-analysis: intervention duration, frequency, period, participant age, and regional income. Among these elements, intervention duration, frequency, participant age, and regional income displayed varying levels of heterogeneity, signifying differential impacts on the exercise intervention’s effect on mental health. In contrast, the cycle subgroup exhibited lower heterogeneity, suggesting that the exercise cycle did not influence the mental health effect.

**Figure 10:**
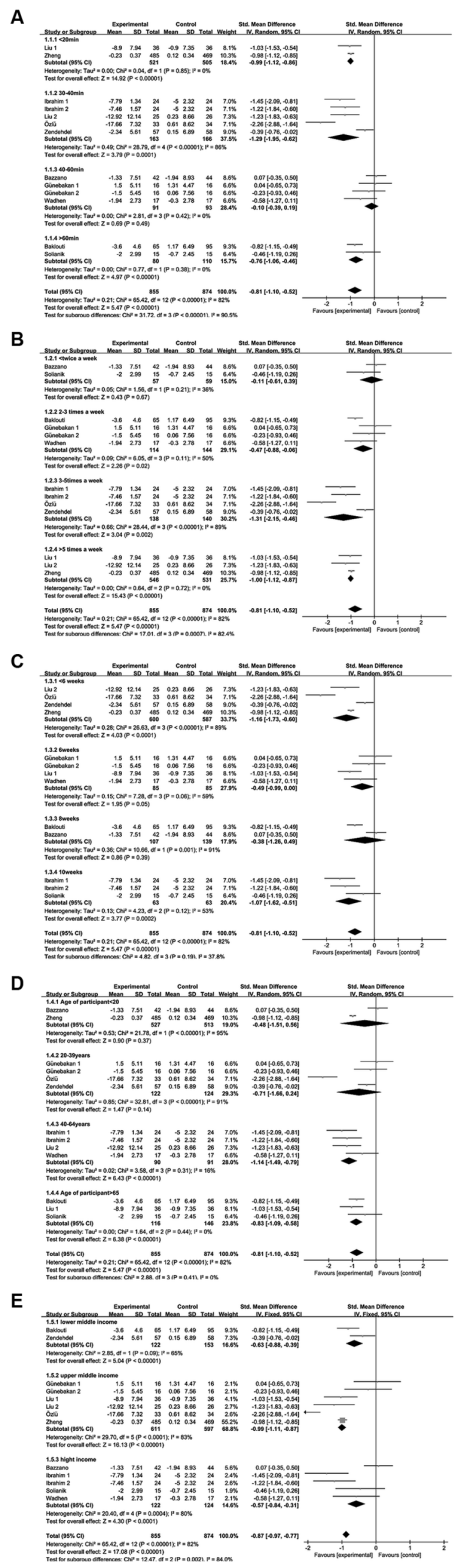
Anxiety subgroup analysis of **(A)** single intervention duration, **(B)** exercise frequency, **(C)** intervention period, **(D)** participant age, and **(E)** income level.

Regarding the duration of single interventions—less than 20 min, 30–40 min, 40–60 min, and more than 60 min—significant heterogeneity in intervention effects was observed among the four groups (I^2^ = 90.5%, *p* < 0.000001), implying the vital role of intervention duration in influencing exercise’s impact on anxiety disorder interventions. The intervention effects on anxiety disorders followed this pattern: less than 20 min (SMD = −0.99), 30–40 min (SMD = −1.29), 40–60 min (SMD = −0.10), and more than 60 min (SMD = −0.76). Statistically significant differences were found in the less than 20 min, 30–40 min, and more than 60 min groups (*p* < 0.05), whereas the 40–60 min group exhibited no statistical significance (*p* = 0.49). Notably, intervention effects for the 30–40 min group surpassed those of the other groups (see Figure 10A).

Examining exercise frequency—less than 2 times per week, 2–3 times per week, 3–5 times per week, and more than 5 times per week—revealed heterogeneous intervention effects among these groups (*I*^2^ = 82.4%, *p* = 0.0007), indicating that intervention frequency influences exercise’s impact on anxiety disorder interventions. The intervention effect sizes for anxiety were as follows: less than 2 sessions per week (SMD = −0.11), 2–3 sessions per week (SMD = −0.47), 3–5 sessions per week (SMD = −1.31), and more than 5 sessions per week (SMD = −1.00). No statistical significance was observed in the less than 2 times per week group (*p* = 0.67), whereas statistical significance emerged in the 2–3 times per week (*p* = 0.02), 3–5 times per week (*p* = 0.002), and more than 5 times per week (*p* < 0.00001) groups. Overall, an intervention frequency of 3–5 sessions per week yielded the most significant improvement in anxiety disorders (refer to Figure 10B).

Analyzing the intervention period—less than 6 weeks, 6 weeks, 8 weeks, and 10 weeks—revealed slight heterogeneity in intervention effect sizes among these groups (*I*^2^ = 37.8%, *p* = 0.19), indicating that the intervention period did not exert a significant influence on the effect of exercise on anxiety disorders (refer to Figure 10C).

Exploring participants’ ages—less than 20 years, 20–39 years, 40–64 years, and more than 65 years—revealed no significant heterogeneity in intervention effect sizes across these groups (*I*^2^ = 0%, *p* = 0.41). This suggests that participants’ age did not exert a significant influence on the impact of exercise and movement interventions for anxiety disorders (refer to Figure 10D).

Examining income levels—low-middle income, high-middle income, and high income—revealed significant heterogeneity in intervention effect sizes across the three groups (*I*^2^ = 82.4%, *p* = 0.002). This underscores income level as a vital determinant affecting the efficacy of exercise interventions for anxiety disorders. The intervention effect sizes for income levels on anxiety disorders were statistically significant (*p* < 0.05) for low-middle income (SMD = −0.63), high-middle income (SMD = −0.99), and high income (SMD = −0.57). Notably, the middle and upper-income groups exhibited notably superior intervention effects (refer to Figure 10E).

**Figure 11:**
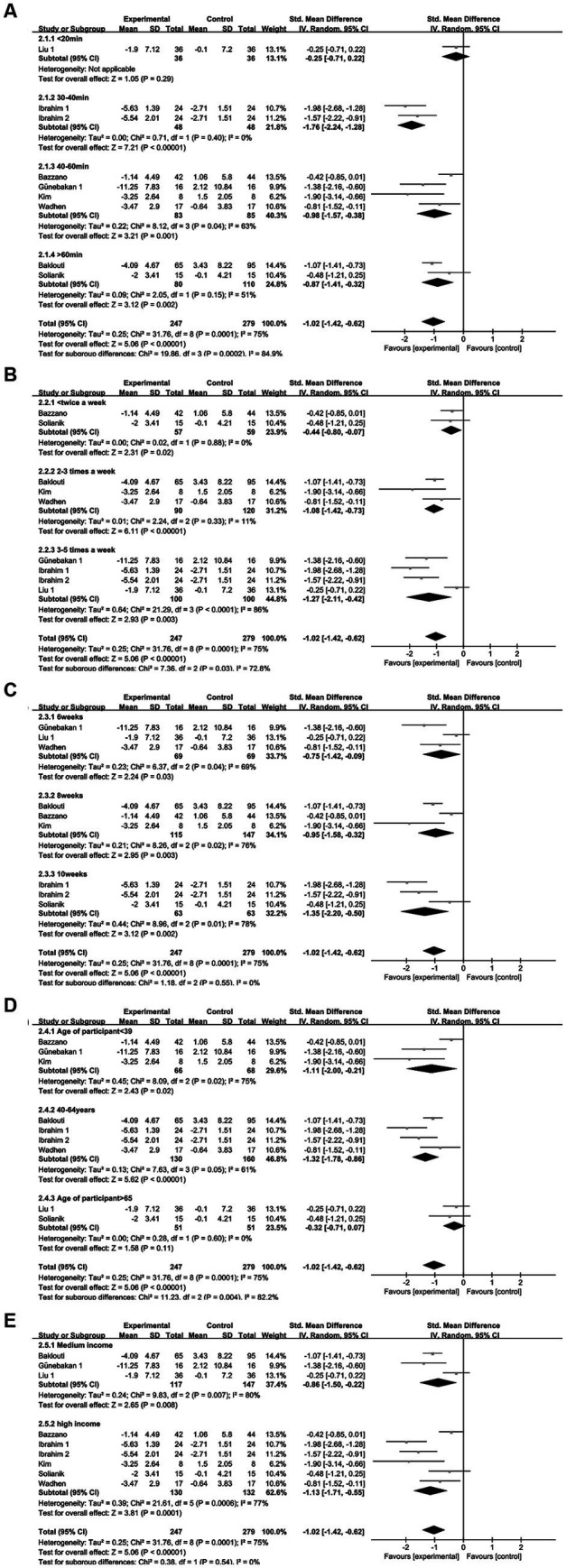
Depression subgroup analysis of **(A)** single intervention duration, **(B)** exercise frequency, **(C)** intervention period, **(D)** participant age, **(E)** income level.

Regarding single intervention duration—less than 20 min, 30–40 min, 40–60 min, and more than 60 min—significant heterogeneity in intervention effect sizes was observed among the four groups (*I*^2^ = 84.9%, *p* = 0.0002). This underscores the pivotal role of intervention duration in influencing the impact of exercise and sports activities on depression interventions. The intervention effect on depression in each group followed this sequence: less than 20 min group (SMD = −0.25), 30–40 min group (SMD = −1.76), 40–60 min group (SMD = −0.98), and more than 60 min group (SMD = −0.87). Statistically significant differences were found in the 30–40 min, 40–60 min, and more than 60 min groups (*p* < 0.05), while no statistical significance emerged in the less than 20 min group (*p* = 0.29). Notably, the 30–40 min group demonstrated notably superior intervention effects compared to the other groups (refer to Figure 11A).

Regarding exercise frequency—less than 2 times per week, 2–3 times per week, and 3–5 times per week—variance in intervention effect sizes was observed among the three groups (*I*^2^ = 72.8%, *p* = 0.03), implying that intervention frequency impacts the efficacy of exercise workouts as a depression intervention. The intervention effect sizes for depression were as follows: less than 2 sessions per week (SMD = −0.44), 2–3 sessions per week (SMD = −1.08), and 3–5 sessions per week (SMD = −1.27), all of which were statistically significant (*p* < 0.05). Notably, the subgroup effect sizes were greater for 2–3 sessions per week and 3–5 sessions per week, with the frequency of 3–5 sessions per week yielding the most substantial intervention effect size for exercise in improving depression (refer to Figure 11B).

Regarding the intervention period—6 weeks, 8 weeks, and 10 weeks—no heterogeneity in intervention effect sizes was observed among the three groups (*I*^2^ = 0%, *p* = 0.55). This indicates that the intervention period does not significantly impact the efficacy of motor exercise interventions for depression (refer to Figure 11C).

Regarding participants’ age—less than 39 years, 40–64 years, and more than 65 years—significant heterogeneity in intervention effect sizes was observed among the three groups (*I*^2^ = 82.2%, *p* = 0.004). This indicates that participants’ age is an influential factor affecting the impact of exercise interventions on depression. The intervention effect sizes were as follows: SMD = −1.11 for the less than 39 years group, SMD = −1.32 for the 40-64-year-old group, and SMD = −0.32 for the more than 65 years group. Statistically significant differences were found in the less than 39-year-old and 40-64-year-old groups (*p* < 0.05), while no statistical significance emerged in the more than 65-year-old group (*p* = 0.11). Notably, the most substantial intervention effect size for exercise in improving depression was observed in the 40-64-year-old group (refer to Figure 11D).

Income level: middle-income and high-income. No heterogeneity in intervention effect sizes was observed between the two groups (*I*^2^ = 0%, *p* = 0.54), implying that income level does not significantly influence the impact of exercise interventions on depression (refer to Figure 11E).

## 4. Discussion

### 4.1. Summarize the main results of the article

The COVID-19 pandemic precipitated a surge in mental health issues. Yet, there is a lack of a systematic assessment and meta-analysis of exercise’s impact on mental health amid the pandemic. This meta-analysis furnishes evidence that exercise during the pandemic ameliorated anxiety disorders, depression, stress levels, and quality of life compared to controls. We assessed 12 eligible studies exploring exercise effects on anxiety, depression, perceived stress, and quality of life during COVID-19. We systematically evaluated these studies, extracting data on sample characteristics, study design, methodological aspects, and mental health outcomes. However, as the included studies were RCTs of exercise interventions, full blinding was unattainable. Previous research suggests potential bias risks even with optimal methods in trials. Nonetheless, deeming trials of low quality solely due to the lack of blinding is not warranted. Consequently, nine studies were deemed of high quality in the assessment process, bolstering our study’s credibility and validity and thereby enhancing the validity and accuracy of our results and conclusions. Even when grouping anxiety and depression outcomes by participant age, intervention frequency, duration, and period, complete heterogeneity elimination wasn’t achieved. Given the diverse interventions and measurement instruments across trials, substantial heterogeneity is unsurprising in this context.

### 4.2. Analysis of the effects of exercise program interventions

We observed significant variations in exercise interventions, encompassing diverse exercise intensities, single exercise durations, exercise frequencies, intervention cycles, and exercise patterns. Initially, by focusing on exercise intensity, its role as an effective treatment method for individuals becomes evident. This aspect holds crucial significance in understanding the direct therapeutic impacts of exercise on mental well-being. However, this pivotal matter remains underexplored in the existing literature. For instance, Gordon BR conducted a meta-analysis of resistance exercise interventions for anxiety disorders (34), scrutinizing the distinction between moderate and high-intensity exercise, thereby critically assessing the optimal resistance exercise dose necessary for anxiety improvement. Nevertheless, this study exclusively centered on resistance exercise, with stricter control over movement patterns. In another investigation, Broman-Fulks JJ demonstrated (35) that low-intensity exercise reduces sensitivity to mental health concerns, while high-intensity exercise amplifies this sensitivity. Our included studies span the COVID-19 pandemic period, and most of them omit reporting exercise intensity. Only two studies provided insight into exercise intensity effects: one study (31) set the intensity at 50–60% of maximal heart rate, while another study (32) compared moderate and low intensity, highlighting the superiority of moderate-intensity aerobic exercise in enhancing exercise capacity, quality of life, and psychological well-being. Merely two studies addressed the influence of exercise intensity on mental health, underscoring the need for further research to ascertain optimal exercise dosages.

Another notable factor is the varying duration of single exercises. The study’s findings highlight that the 30-40-min single exercise duration group exhibited the most pronounced impact on alleviating anxiety and depression, markedly surpassing the groups with durations of less than 20 min, 40–60 min, and more than 60 min. Notably, intervention effect sizes declined with extended exercise durations. This trend aligns with the World Health Organization’s recommendation of 30–60 min of moderate-intensity aerobic exercise per session. In the context of the COVID-19 pandemic, Peijie’s investigation (36) revealed that excessively long exercise sessions induced additional fatigue and stress among participants.

An additional variable to consider is exercise frequency, which exhibits varying impacts on mental health. The findings indicated that an exercise frequency of 3–5 times per week yielded the most significant improvements in anxiety and depression, aligning with the World Health Organization’s recommendation of 3 times per week. Notably, as the intervention frequency increased or decreased, the corresponding intervention effects diminished. Chekroud (37) conducted a substantial cross-sectional study on the correlation between physical activity and mental health, involving around 1 to 2 million individuals in the United States. This research identified (38) the efficacy of three to five exercise sessions per week in alleviating psychological burdens. Moreover, Chekroud’s study (37) underscored that a frequency surpassing 5 interventions per week might hinder full muscle recovery, potentially leading to exercise load accumulation and subsequent immune system, psychological, and sleep disorders. Conversely, exercise frequencies below 2 times per week demonstrated relatively lower effectiveness in addressing anxiety, depression, and related mental health issues (39).

The impact of distinct intervention cycles represents the fourth variable under scrutiny. Our findings reveal significant effects across short and long cycles, yet no substantial disparities emerged. Consequently, the intervention period’s influence on the effectiveness of exercise interventions for anxiety and depression appears minimal. This outcome could be attributed to potential counteracting factors inherent to the diverse study designs, methodologies, and participant characteristics. Among these factors, individual physiological adaptations and psychological responses to exercise exhibit variations due to inherent differences (40). Certain individuals might favor short-term, high-intensity exercise, while others could benefit more from extended periods of low-intensity activity. These individual variances tend to balance out to some degree, mitigating potential differences on a broader scale. Thus, the comparable effects of diverse exercise cycles on anxiety and depression interventions yielded no significant overall discrepancies.

The fifth aspect under examination is exercise patterns. Notably, amidst the COVID-19 pandemic, no studies were identified that directly compared the impacts of varied exercise patterns or types on mental health. Nevertheless, it is important to highlight that all exercise modalities exhibited a reduction in mental health burden. Among the studies encompassed in this review, three (25, 28, 29) incorporated progressive muscle relaxation exercises, while four (20, 27, 30, 33) focused on yoga–both of which are categorized as aerobic exercises. It is worth noting that no investigations about the effects of resistance exercise on mental health during the COVID-19 pandemic were found, thus precluding a conclusive assessment of diverse exercise patterns.

### 4.3. Analysis of the effect of age and income level

Besides variations in exercise intervention programs, disparities in age and income level were observed to impact the distinct outcomes of exercise interventions on mental health. Initially, the influence of age on exercise intervention efficacy for mental health was explored. The findings from this investigation indicated that participants’ age did not significantly affect the effectiveness of exercise interventions for anxiety disorders. However, in the context of depression, participant age emerged as a pivotal factor influencing the potency of sport and exercise interventions. Particularly, exercise exhibited its most pronounced impact on alleviating depression within the 40–64 years age group, a phase categorized as midlife according to the International Health Organization’s age classification. Midlife, spanning 40 to 64 years, represents a phase of heightened vulnerability to mental health challenges. Kessler’s research (41) illuminated that during midlife, individuals often encounter a confluence of stressors encompassing occupational pressures, familial responsibilities, and life transitions, amplifying their susceptibility to mental health issues. The intrinsic stresses and shifts characteristic of middle adulthood render individuals more predisposed to depressive symptoms, thereby augmenting the incidence of depression within this age bracket. Notably, a systematic evaluation (42) and a meta-analysis (43) have corroborated the pivotal role of exercise in effectively intervening against depression among middle-aged adults by enhancing physiological well-being, facilitating social engagements, and augmenting psychological states.

Next, the impact of income level on exercise intervention’s influence on mental health is examined. The study outcomes suggest that income level does not significantly affect the efficacy of exercise interventions for depression. However, noteworthy improvements in anxiety were observed within the upper-middle-income group. Amid the COVID-19 pandemic, numerous coping barriers surfaced, encompassing food insecurity, mental health challenges, isolation, economic strain, restricted healthcare access, and virtual schooling, further exacerbated by limited Internet access. Enhanced social support and networking resources often characterize middle- and high-income regions, offering online and offline avenues for health promotion and exercise (44). This expanded resource spectrum provides individuals with diverse options, rendering exercise a feasible approach to alleviating anxiety symptoms. Additionally, middle- and upper-income neighborhoods host a greater number of profit-driven establishments that facilitate physical activity opportunities (45), contributing to a reduced prevalence of physical inactivity (46). The heightened physical activity levels in upper-middle-income regions hold the potential to enhance mental health, avert chronic ailments, and elevate overall quality of life.

### 4.4. Mechanisms of the effect of exercise on mental health during the COVID-19 pandemic

The COVID-19 pandemic triggers a systemic and chronic inflammatory response, with inflammatory molecules potentially entering the brain and disrupting neurotransmitter equilibrium, subsequently affecting mood and cognitive functions (47, 48). The pandemic-induced tension and uncertainty may further disturb neurotransmitter balance (49, 50). Factors like chronic health concerns, social instability, and economic stress can exacerbate persistent stress responses (51, 52). Although limited, research has begun exploring the mechanisms underlying the relationship between exercise and mental health, identifying several potential pathways. First, there’s a physiological perspective. Exercise can alleviate anxiety, depression, and stress by releasing endogenous hormones (e.g., endorphins, dopamine, and serotonin) with analgesic and pleasurable effects. Physical activity aids in neurotransmitter regulation (e.g., 24-hydroxytryptophan, norepinephrine), fostering neuroplasticity and proper brain region functioning, thereby enhancing positive moods (53–55). Additionally, exercise assists in modulating the hypothalamic–pituitary–adrenal (HPA) axis function, curbing excessive stress responses, and mitigating anxiety and depression symptoms (43, 56). Moreover, exercise stimulates the immune system to release beneficial cytokines, particularly anti-inflammatory ones, fostering immune balance and curbing excessive inflammation–a crucial component for mental well-being (57, 58). Second, a social-behavioral explanation exists. Exercise can elevate an individual’s self-esteem and self-efficacy, enabling more constructive coping with mental health issues (59). The attainment of physical activity goals brings forth a sense of achievement, positively impacting emotional states. Furthermore, exercise serves as a platform for social interaction, diminishing social isolation and augmenting mental health (60).

### 4.5. Limitations and advantages

This systematic evaluation and meta-analysis have limitations. Firstly, the included studies were randomized controlled trials of exercise interventions, precluding complete blinding. Thus, subjective factors might introduce bias into the quality assessment process. Secondly, non-uniform scales employed to evaluate mental health indicators led to considerable heterogeneity. Thirdly, due to the limited follow-up data reported in the included studies, the long-term effects of exercise on COVID-19 pandemic-related mental health were not analyzed. Lastly, despite encompassing multiple countries, the meta-analysis lacked representation from low-income areas.

This study holds numerous strengths. Firstly, it pioneers in systematically evaluating and meta-analyzing the impact of exercise on mental health during the COVID-19 pandemic, introducing an innovative dimension. Secondly, adhering to PRISMA guidelines, our review employed a rigorous systematic methodology, ensuring meticulous identification and assessment of pertinent literature with optimal scientific rigor. Thirdly, our review was *a priori* registered in the Prospero database, predefining research inquiries and inclusion criteria and enhancing methodological transparency. Fourthly, an extensive search across three electronic sources was conducted, documented in our comprehensive search strategy detailed in the electronic Supplementary Table S1. Additionally, the quality of the included studies was scrupulously examined, fortifying the conclusions drawn *via* quality assessment tools. Fifthly, we explored the influence of exercise on COVID-19 pandemic-linked mental health from diverse standpoints, encompassing exercise regimes, age, and regional income levels. Our findings offer valuable insights for the real-time enhancement of mental health services and the elevation of initiatives to foster positive mental health outcomes.

## 5. Conclusion

Our analysis demonstrated the efficacy of exercise in alleviating anxiety disorders, depression, and stress levels and enhancing quality of life among individuals during the COVID-19 pandemic. Notably, a physical activity regimen with a single exercise duration of 30–40 min and an exercise frequency of 3–5 times per week yielded the most pronounced effects in ameliorating anxiety and depression. Particularly, individuals aged 40–64 years experienced the most significant improvements in depression through exercise interventions. Moreover, exercise was most impactful in reducing anxiety among individuals from middle- and high-income backgrounds. For future research, it is imperative to address certain aspects. Firstly, expanding the study sample size, standardizing tools for assessing mental health indicators, and extending exercise cycles will enhance the comprehensiveness of meta-analysis outcomes. Secondly, employing more rigorous and scientifically robust methods to elevate the quality of randomized controlled trials (RCTs) will enable the derivation of more conclusive results. This, in turn, will offer enhanced references for medical practitioners and serve as a valuable scientific foundation for individuals engaging in physical exercise.

## Data availability statement

The original contributions presented in the study are included in the article/Supplementary material, further inquiries can be directed to the corresponding author.

## Author contributions

CW: Data curation, Methodology, Software, Writing – original draft, Writing – review & editing. ZT: Supervision, Writing – review & editing. QL: Data curation, Methodology, Writing – original draft.
