# Supplementary material for: The impact of exercise on mental health during the COVID-19 pandemic: a systematic review and meta-analysis
Source: Front Public Health. 2023 Oct 4;11:1279599. doi: 10.3389/fpubh.2023.1279599 (PMC10582957; doi:10.3389/fpubh.2023.1279599)
Supplement: Supplementary file 1 [file Presentation_1.pdf]

Supplementary Material

1 Search string

1.1 Search string for Pubmed

"COVID-19"[MeSH Terms] OR "COVID-19"[Supplementary Concept] OR "COVID-19"[All Fields] OR "2019 ncov infection"[All Fields] OR "COVID-19"[MeSH Terms] OR "COVID-19"[Supplementary Concept] OR "COVID-19"[All Fields] OR "2019 ncov infection"[All Fields] OR "COVID-19"[MeSH Terms] OR "COVID-19"[Supplementary Concept] OR "COVID-19"[All Fields] OR "2019 ncov infections"[All Fields] OR "COVID-19"[MeSH Terms] OR "COVID-19"[Supplementary Concept] OR "COVID-19"[All Fields] OR "infection 2019 ncov"[All Fields] OR "COVID-19"[MeSH Terms] OR "COVID-19"[Supplementary Concept] OR "COVID-19"[All Fields] OR "sars cov 2 infection"[All Fields] OR "COVID-19"[MeSH Terms] OR "COVID-19"[Supplementary Concept] OR "COVID-19"[All Fields] OR "infection sars cov 2"[All Fields] OR "COVID-19"[MeSH Terms] OR "COVID-19"[Supplementary Concept] OR "COVID-19"[All Fields] OR "sars cov 2 infection"[All Fields] OR "COVID-19"[MeSH Terms] OR "COVID-19"[Supplementary Concept] OR "COVID-19"[All Fields] OR "sars cov 2 infections"[All Fields] OR "COVID-19"[MeSH Terms] OR "COVID-19"[Supplementary Concept] OR "COVID-19"[All Fields] OR "2019 novel coronavirus disease"[All Fields] OR "COVID-19"[MeSH Terms] OR "COVID-19"[Supplementary Concept] OR "COVID-19"[All Fields] OR "2019 novel coronavirus infection"[All Fields] OR "COVID-19"[MeSH Terms] OR "COVID-19"[Supplementary Concept] OR "COVID-19"[All Fields] OR "covid 19 virus infection"[All Fields] OR "COVID-19"[MeSH Terms] OR "COVID-19"[Supplementary Concept] OR "COVID-19"[All Fields] OR "covid 19 virus infection"[All Fields] OR "COVID-19"[MeSH Terms] OR "COVID-19"[Supplementary Concept] OR "COVID-19"[All Fields] OR "covid 19 virus infections"[All Fields] OR "COVID-19"[MeSH Terms] OR "COVID-19"[Supplementary Concept] OR "COVID-19"[All Fields] OR "infection covid 19 virus"[All Fields] OR "COVID-19"[MeSH Terms] OR "COVID-19"[Supplementary Concept] OR "COVID-19"[All Fields] OR "virus infection covid 19"[All Fields] OR "COVID-19"[MeSH Terms] OR "COVID-19"[Supplementary Concept] OR "COVID-19"[All Fields] OR "covid19"[All Fields] OR "COVID-19"[MeSH Terms] OR "COVID-19"[Supplementary Concept] OR "COVID-19"[All Fields] OR "coronavirus disease 2019"[All Fields] OR "COVID-19"[MeSH Terms] OR "COVID-19"[Supplementary Concept] OR "COVID-19"[All Fields] OR "disease 2019 coronavirus"[All Fields] OR "COVID-19"[MeSH Terms] OR "COVID-19"[Supplementary Concept] OR "COVID-19"[All Fields] OR "coronavirus disease 19"[All Fields] OR "COVID-19"[MeSH Terms] OR "COVID-19"[Supplementary Concept] OR "COVID-19"[All Fields] OR "coronavirus disease 19"[All Fields] OR "COVID-19"[MeSH Terms] OR "COVID-19"[Supplementary Concept] OR "COVID-19"[All Fields] OR "severe acute respiratory syndrome coronavirus 2 infection"[All Fields] OR "COVID-19"[MeSH Terms] OR "COVID-19"[Supplementary Concept] OR "COVID-19"[All Fields] OR "covid 19 virus disease"[All Fields] OR "COVID-19"[MeSH Terms] OR "COVID-19"[Supplementary Concept] OR "COVID-19"[All Fields] OR "covid 19 virus disease"[All Fields] OR "COVID-19"[MeSH Terms] OR "COVID-19"[Supplementary Concept] OR "COVID-19"[All Fields] OR "COVID-19"[MeSH Terms] OR "COVID-19"[Supplementary Concept] OR "COVID-19"[All Fields] OR "disease covid 19 virus"[All Fields] OR "COVID-19"[MeSH Terms] OR "COVID-19"[Supplementary Concept] OR "COVID-19"[All Fields] OR "virus disease covid 19"[All

Fields] OR "COVID-19"[MeSH Terms] OR "COVID-19"[Supplementary Concept] OR "COVID-19"[All Fields] OR "sars coronavirus 2 infection"[All Fields] OR "COVID-19"[MeSH Terms] OR "COVID-19"[Supplementary Concept] OR "COVID-19"[All Fields] OR "2019 ncov disease"[All Fields] OR "COVID-19"[MeSH Terms] OR "COVID-19"[Supplementary Concept] OR "COVID-19"[All Fields] OR "2019 ncov disease"[All Fields] OR "COVID-19"[MeSH Terms] OR "COVID-19"[Supplementary Concept] OR "COVID-19"[All Fields] OR "2019 ncov diseases"[All Fields] OR "COVID-19"[MeSH Terms] OR "COVID-19"[Supplementary Concept] OR "COVID-19"[All Fields] OR "disease 2019 ncov"[All Fields] OR "COVID-19"[MeSH Terms] OR "COVID-19"[Supplementary Concept] OR "COVID-19"[All Fields] OR "covid 19 pandemic"[All Fields] OR "COVID-19"[MeSH Terms] OR "COVID-19"[Supplementary Concept] OR "COVID-19"[All Fields] OR "covid 19 pandemic"[All Fields] OR "COVID-19"[MeSH Terms] OR "COVID-19"[Supplementary Concept] OR "COVID-19"[All Fields] OR "pandemic covid 19"[All Fields] OR "COVID-19"[MeSH Terms] OR "COVID-19"[Supplementary Concept] OR "COVID-19"[All Fields] OR "covid 19 pandemics"[All Fields] OR "COVID-19"[MeSH Terms]

AND

"Exercise"[MeSH Terms] OR ("Exercise"[MeSH Terms] OR "Exercise"[All Fields] OR "exercises"[All Fields] OR "exercise therapy"[MeSH Terms] OR ("Exercise"[All Fields] AND "therapy"[All Fields]) OR "exercise therapy"[All Fields] OR "exercising"[All Fields] OR "exercise s"[All Fields] OR "exercised"[All Fields] OR "exerciser"[All Fields] OR "exercisers"[All Fields] OR ("Exercise"[MeSH Terms] OR "Exercise"[All Fields] OR ("physical"[All Fields] AND "activity"[All Fields]) OR "physical activity"[All Fields]) OR ("Exercise"[MeSH Terms] OR "Exercise"[All Fields] OR ("activities"[All Fields] AND "physical"[All Fields]) OR "activities physical"[All Fields]) OR ("Exercise"[MeSH Terms] OR "Exercise"[All Fields] OR ("activity"[All Fields] AND "physical"[All Fields]) OR "activity physical"[All Fields]) OR ("Exercise"[MeSH Terms] OR "Exercise"[All Fields] OR ("physical"[All Fields] AND "activities"[All Fields]) OR "physical activities"[All Fields]) OR ("Exercise"[MeSH Terms] OR "Exercise"[All Fields] OR ("Exercise"[All Fields] AND "physical"[All Fields]) OR "exercise physical"[All Fields]) OR ("Exercise"[MeSH Terms] OR "Exercise"[All Fields] OR ("exercises"[All Fields] AND "physical"[All Fields]) OR "exercises physical"[All Fields]) OR ("Exercise"[MeSH Terms] OR "Exercise"[All Fields] OR ("physical"[All Fields] AND "Exercise"[All Fields]) OR "physical exercise"[All Fields]) OR ("Exercise"[MeSH Terms] OR "Exercise"[All Fields] OR ("physical"[All Fields] AND "exercises"[All Fields]) OR "physical exercises"[All Fields]) OR ("Exercise"[MeSH Terms] OR "Exercise"[All Fields] OR ("acute"[All Fields] AND "Exercise"[All Fields]) OR "acute exercise"[All Fields]) OR ("Exercise"[MeSH Terms] OR "Exercise"[All Fields] OR ("acute"[All Fields] AND "exercises"[All Fields]) OR "acute exercises"[All Fields]) OR ("Exercise"[MeSH Terms] OR "Exercise"[All Fields] OR ("Exercise"[All Fields] AND "acute"[All Fields]) OR "exercise acute"[All Fields]) OR ("Exercise"[MeSH Terms] OR "Exercise"[All Fields] OR ("exercises"[All Fields] AND "acute"[All Fields]) OR "exercises acute"[All Fields]) OR ("Exercise"[MeSH Terms] OR "Exercise"[All Fields] OR ("Exercise"[All Fields] AND "isometric"[All Fields]) OR "exercise isometric"[All Fields]) OR ("Exercise"[MeSH Terms] OR "Exercise"[All Fields] OR ("exercises"[All Fields] AND "isometric"[All Fields]) OR "exercises isometric"[All Fields]) OR ("Exercise"[MeSH Terms] OR "Exercise"[All Fields] OR ("isometric"[All Fields] AND "exercises"[All Fields]) OR "isometric exercises"[All Fields]) OR ("Exercise"[MeSH Terms] OR "Exercise"[All Fields] OR ("isometric"[All Fields] AND "Exercise"[All Fields]) OR "isometric exercise"[All Fields]) OR ("Exercise"[MeSH Terms] OR "Exercise"[All Fields] OR ("Exercise"[All Fields] AND "aerobic"[All

Fields]) OR "exercise aerobic"[All Fields]) OR ("Exercise"[MeSH Terms] OR "Exercise"[All Fields] OR ("aerobic"[All Fields] AND "Exercise"[All Fields]) OR "aerobic exercise"[All Fields]) OR ("Exercise"[MeSH Terms] OR "Exercise"[All Fields] OR ("aerobic"[All Fields] AND "exercises"[All Fields]) OR "aerobic exercises"[All Fields]) OR ("Exercise"[MeSH Terms] OR "Exercise"[All Fields] OR ("exercises"[All Fields] AND "aerobic"[All Fields]) OR "exercises aerobic"[All Fields]) OR ("Exercise"[MeSH Terms] OR "Exercise"[All Fields] OR ("Exercise"[All Fields] AND "training"[All Fields]) OR "exercise training"[All Fields]) OR ("Exercise"[MeSH Terms] OR "Exercise"[All Fields] OR ("Exercise"[All Fields] AND "trainings"[All Fields]) OR "exercise trainings"[All Fields]) OR ("Exercise"[MeSH Terms] OR "Exercise"[All Fields] OR ("training"[All Fields] AND "Exercise"[All Fields]) OR "training exercise"[All Fields]) OR ("Exercise"[MeSH Terms] OR "Exercise"[All Fields] OR ("trainings"[All Fields] AND "Exercise"[All Fields])))

AND

"Mental Health"[MeSH Terms] OR ("Mental Health"[MeSH Terms] OR ("mental"[All Fields] AND "health"[All Fields]) OR "Mental Health"[All Fields] OR ("Mental Health"[MeSH Terms] OR ("mental"[All Fields] AND "health"[All Fields]) OR "Mental Health"[All Fields] OR ("health"[All Fields] AND "mental"[All Fields]) OR "health mental"[All Fields]) OR ("Mental Health"[MeSH Terms] OR ("mental"[All Fields] AND "health"[All Fields]) OR "Mental Health"[All Fields] OR ("mental"[All Fields] AND "hygiene"[All Fields]) OR "mental hygiene"[All Fields]) OR ("Mental Health"[MeSH Terms] OR ("mental"[All Fields] AND "health"[All Fields]) OR "Mental Health"[All Fields] OR ("hygiene"[All Fields] AND "mental"[All Fields]) OR "hygiene, mental"[All Fields]))

1.2 Search string for Web of Science

(TS=(COVID-19) OR TS=(COVID 19) OR TS=(2019-nCoV Infection) OR TS=(2019 nCoV Infection) OR TS=(2019-nCoV Infections) OR TS=(Infection, 2019-nCoV) OR TS=(SARS-CoV-2 Infection) OR TS=(Infection, SARS-CoV-2) OR TS=(SARS CoV 2 Infection) OR TS=(SARS-CoV-2 Infections) OR TS=(2019 Novel Coronavirus Disease) OR TS=(2019 Novel Coronavirus Infection) OR TS=(COVID-19 Virus Infection) OR TS=(COVID 19 Virus Infection) OR TS=(COVID-19 Virus Infections) OR TS=(Infection, COVID-19 Virus) OR TS=(Virus Infection, COVID-19) OR TS=(COVID19) OR TS=(Coronavirus Disease 2019) OR TS=(Disease 2019, Coronavirus) OR TS=(Coronavirus Disease-19) OR TS=(Coronavirus Disease 19) OR TS=(Severe Acute Respiratory Syndrome Coronavirus 2 Infection) OR TS=(COVID-19 Virus Disease) OR TS=(COVID 19 Virus Disease) OR TS=(COVID-19 Virus Diseases) OR TS=(Disease, COVID-19 Virus) OR TS=(Virus Disease, COVID-19) OR TS=(SARS Coronavirus 2 Infection) OR TS=(2019-nCoV Disease) OR TS=(2019 nCoV Disease) OR TS=(2019-nCoV Diseases) OR TS=(Disease, 2019-nCoV) OR TS=(COVID-19 Pandemic) OR TS=(COVID 19 Pandemic) OR TS=(Pandemic, COVID-19) OR TS=(COVID-19 Pandemics)) NOT (SILOID==("PPRN"))

AND

(TS=(Exercises) OR TS=(Physical Activity) OR TS=(Activities, Physical) OR TS=(Activity, Physical) OR TS=(Physical Activities) OR TS=(Exercise, Physical) OR TS=(Exercises, Physical) OR TS=(Physical Exercise) OR TS=(Physical Exercises) OR TS=(Acute Exercise) OR TS=(Acute Exercises) OR TS=(Exercise, Acute) OR TS=(Exercises, Acute) OR TS=(Exercise, Isometric) OR TS=(Exercises, Isometric) OR TS=(Isometric Exercises) OR TS=(Isometric Exercise) OR TS=(Exercise, Aerobic) OR TS=(Aerobic Exercise) OR TS=(Aerobic Exercises) OR TS=(Exercises,

Aerobic) OR TS=(Exercise Training) OR TS=(Exercise Trainings) OR TS=(Training, Exercise) OR TS=(Trainings, Exercise)) NOT (SILOID==("PPRN"))

AND

(TS=(Mental health) OR TS=(Health, Mental) OR TS=(Mental Hygiene) OR TS=(Hygiene, Mental)) NOT (SILOID==("PPRN"))

1.3 Search string for SCOPUS

(TITLE-ABS-KEY(COVID-19) OR TITLE-ABS-KEY(COVID 19) OR TITLE-ABS-KEY(2019-nCoV Infection) OR TITLE-ABS-KEY(2019 nCoV Infection) OR TITLE-ABS-KEY(2019-nCoV Infections) OR TITLE-ABS-KEY(Infection, 2019-nCoV) OR TITLE-ABS-KEY(SARS-CoV-2 Infection) OR TITLE-ABS-KEY(Infection, SARS-CoV-2) OR TITLE-ABS-KEY(SARS CoV 2 Infection) OR TITLE-ABS-KEY(SARS-CoV-2 Infections) OR TITLE-ABS-KEY(2019 Novel Coronavirus Disease) OR TITLE-ABS-KEY(2019 Novel Coronavirus Infection) OR TITLE-ABS-KEY(COVID-19 Virus Infection) OR TITLE-ABS-KEY(COVID 19 Virus Infection) OR TITLE-ABS-KEY(COVID-19 Virus Infections) OR TITLE-ABS-KEY(Infection, COVID-19 Virus) OR TITLE-ABS-KEY(Virus Infection, COVID-19) OR TITLE-ABS-KEY(COVID19) OR TITLE-ABS-KEY(Coronavirus Disease 2019) OR TITLE-ABS-KEY(Disease 2019, Coronavirus) OR TITLE-ABS-KEY(Coronavirus Disease-19) OR TITLE-ABS-KEY(Coronavirus Disease 19) OR TITLE-ABS-KEY(Severe Acute Respiratory Syndrome Coronavirus 2 Infection) OR TITLE-ABS-KEY(COVID-19 Virus Disease) OR TITLE-ABS-KEY(COVID 19 Virus Disease) OR TITLE-ABS-KEY(COVID-19 Virus Diseases) OR TITLE-ABS-KEY(Disease, COVID-19 Virus) OR TITLE-ABS-KEY(Virus Disease, COVID-19) OR TITLE-ABS-KEY(SARS Coronavirus 2 Infection) OR TITLE-ABS-KEY(2019-nCoV Disease) OR TITLE-ABS-KEY(2019 nCoV Disease) OR TITLE-ABS-KEY(2019-nCoV Diseases) OR TITLE-ABS-KEY(Disease, 2019-nCoV) OR TITLE-ABS-KEY(COVID-19 Pandemic) OR TITLE-ABS-KEY(COVID 19 Pandemic) OR TITLE-ABS-KEY(Pandemic, COVID-19) OR TITLE-ABS-KEY(COVID-19 Pandemics))

AND

(TITLE-ABS-KEY(Exercises) OR TITLE-ABS-KEY(Physical Activity) OR TITLE-ABS-KEY(Activities, Physical) OR TITLE-ABS-KEY(Activity, Physical) OR TITLE-ABS-KEY(Physical Activities) OR TITLE-ABS-KEY(Exercise, Physical) OR TITLE-ABS-KEY(Exercises, Physical) OR TITLE-ABS-KEY(Physical Exercise) OR TITLE-ABS-KEY(Physical Exercises) OR TITLE-ABS-KEY(Acute Exercise) OR TITLE-ABS-KEY(Acute Exercises) OR TITLE-ABS-KEY(Exercise, Acute) OR TITLE-ABS-KEY(Exercises, Acute) OR TITLE-ABS-KEY(Exercise, Isometric) OR TITLE-ABS-KEY(Exercises, Isometric) OR TITLE-ABS-KEY(Isometric Exercises) OR TITLE-ABS-KEY(Isometric Exercise) OR TITLE-ABS-KEY(Exercise, Aerobic) OR TITLE-ABS-KEY(Aerobic Exercise) OR TITLE-ABS-KEY(Aerobic Exercises) OR TITLE-ABS-KEY(Exercises, Aerobic) OR TITLE-ABS-KEY(Exercise Training) OR TITLE-ABS-KEY(Exercise Trainings) OR TITLE-ABS-KEY(Training, Exercise) OR TITLE-ABS-KEY(Trainings, Exercise))

AND

(TITLE-ABS-KEY(Mental health) OR TITLE-ABS-KEY(Health, Mental) OR TITLE-ABS-KEY(Mental Hygiene) OR TITLE-ABS-KEY(Hygiene, Mental))
